# Supplementary material for: The structure and stability of Fe4+xS3 and its potential to form a Martian inner core
Source: Nat Commun. 2025 Feb 25;16:1710. doi: 10.1038/s41467-025-56220-2 (PMC11861659; doi:10.1038/s41467-025-56220-2)
Supplement: Supplementary file 1 — Supplementary Information [file 41467_2025_56220_MOESM1_ESM.pdf]

Supplementary Information for

The structure and stability of $Fe_{4+x}S_3$ and its potential to form a Martian inner core

Lianjie Man[1*], Xiang Li[2], Tiziana Boffa Ballaran[1], Wenju Zhou[3], Julien Chantel[4], Adrien Néri[1,4], Ilya Kupenko[2], Georgios Aprilis[2], Alexander Kurnosov[1], Olivier Namur[5], Michael Hanfland[2], Nicolas Guignot[6], Laura Henry[6], Leonid Dubrovinsky[1], Daniel. J. Frost[1]

[1]Bayerisches Geoinstitut, Universität Bayreuth, Bayreuth, Germany.

[2]European Synchrotron Radiation Facility, Grenoble, France.

[3]Material Physics and Technology at Extreme Conditions, Laboratory of Crystallography, University of Bayreuth, Bayreuth, Germany.

[4]Univ. Lille, CNRS, INRAE, Centrale Lille, UMR 8207 - UMET - Unité Matériaux et Transformations, F-59000 Lille, France.

[5]Earth and Environmental Sciences, KU Leuven, 3001 Leuven, Belgium.

[6]Synchrotron SOLEIL, L'Orme de Merisiers, Saint Aubin-BP48, 91192 Gif-sur-Yvette, France.

email: lianjie.man@uni-baryreuth.de (Lianjie Man)

Supplementary Methods

Single crystal structure solution and refinement of $Fe_{4+x}S_3$. We utilized the Olex2 software package[1] to solve and refine the single-crystal data after data reduction. Atomic scattering factors were used for the Fe and S atoms. In a preliminary model the structural solution identified four Fe positions (Fe1-Fe4) and three S positions (S1-S3), which form edge-sharing Fe-S square pyramids. All these atomic positions were refined anisotropically. However, significant electron density was observed in the interstitial sites among the square pyramids. The electron cloud intensity was insufficient to be identified as an Fe atom, and if identified as an S atom, the S-S bond length would be unrealistically short. We used the Platon software within the Olex2 package to search unsuccessfully for twin laws, confirming that the abnormal electron density in the interstitial site was not due to twinning. Based on the known compositional information of this phase, we propose that the interstitial site is occupied only partially by Fe (Fe5). Since the occupancies for Fe1, Fe2, Fe3, Fe4, S1, S2, and S3 were 0.98, 1.00, 0.98, 0.98, 1.00, 0.99, and 1.03, respectively, these positions can be considered fully occupied within the measurement error range. Therefore, we fixed these positions to full occupancy and refined the occupancy of the Fe5 position which was refined isotropically.

Supplementary Discussion

Single-crystal structure-chemistry relationship. The two single-crystal samples analyzed in this study have different compositions due to the presence of a different amount of Fe in the tetrahedral interstitial site (Fe5). As the amount of Fe increases at the Fe5 site the tetrahedral volume also increases. Such changes cannot be compared directly, since the pressure at which the two samples have been measured is different, however, since the change in the tetrahedral volume between sample LJFeS01 (4.54(1) $Å^3$, x=0.11) and sample LD101 (4.85(1) $Å^3$, x = 0.77) is 7%, we can expect a much larger difference when compared at a same pressure. The polyhedral volumes of sites Fe1, Fe2, Fe3 and Fe4 also increase due to the opening of the S atoms sub-lattice caused by the increase in amount of Fe at the Fe5 site. Such changes are not uniform because they depend on which S atom belongs to the coordination sphere, and are less than 3% for Fe1, Fe2 and Fe3 but are 7% for Fe4. The effect of composition on the unit-cell volume is shown in Supplementary Fig. 1 where the compression of sample LJFeS01 (x = 0.11) is compared with the unit-cell volume of sample LD101 (x = 0.77), which is approximately 6% larger due to the presence of more Fe into the Fe5 site.

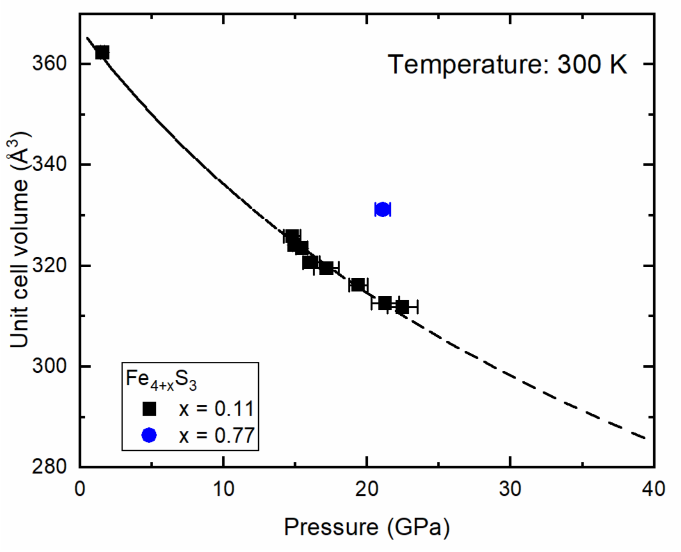

Supplementary Fig. 1 P-V-T-x relations of $Fe_{4+x}S_3$ under high pressure and room temperature. The error bars indicate the uncertainties in pressure, stemming from the inaccuracies in volume determinations of the pressure markers.

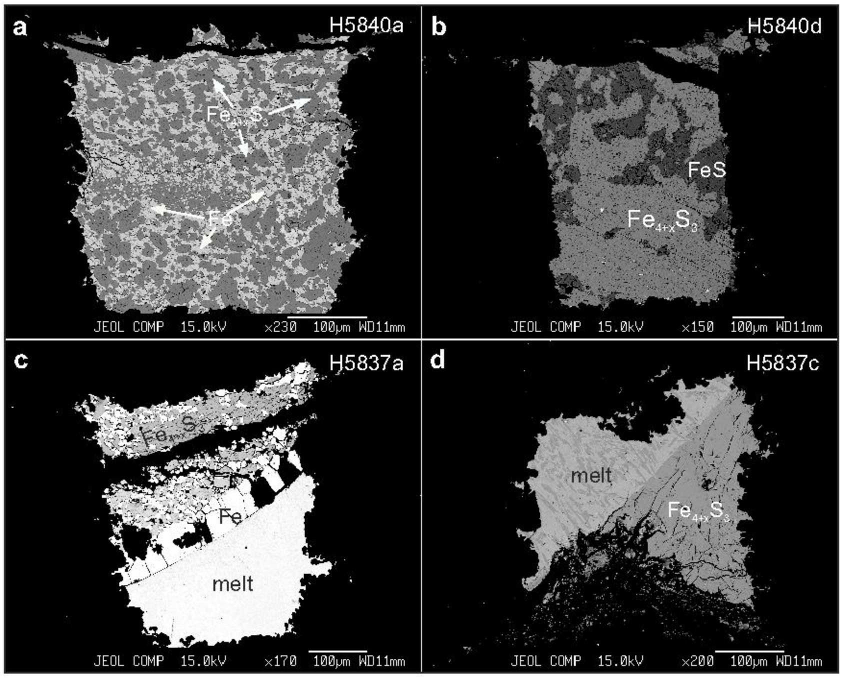

Supplementary Fig. 2 Backscattered electron images depicting $Fe_{4+x}S_3$ and associated phases from multi-anvil experiments, all encapsulated in MgO capsules. a $Fe_{4+x}S_3$ alongside metallic Fe, obtained at 16 GPa and 998 K. **b** $Fe_{4+x}S_3$ with FeS, also from 16 GPa and 998 K. **c** $Fe_{4+x}S_3$ in the presence of metallic iron and a melt phase, recovered from 16 GPa and 1266 K. **d** $Fe_{4+x}S_3$ with a melt phase, recovered from 16 GPa and 1266 K.

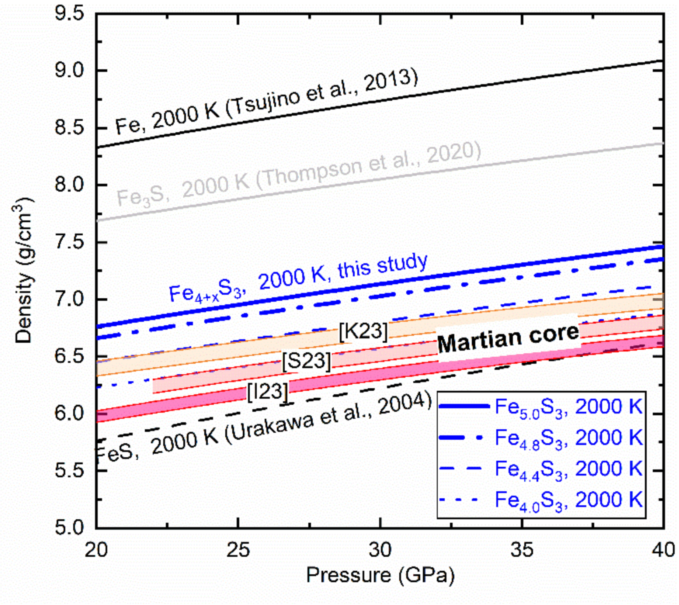

Supplementary Fig. 3 Comparison between the densities of Fe, Fe_3S, $Fe_{4+x}S_3$, and FeS under Martian core conditions and at 2000 K. The blue curves are for $Fe_{4+x}S_3$ determined in this study for x = 0, 0.4, 0.8, and 1. The black solid curve, light grey solid curve, and black dashed curves are the density of fcc iron[2], Fe_3S[3], and FeS[4]. Density estimates for the liquid Martian core (S23: Samuel et al.[5]; I23: Irving et al.[6]; K23: Khan et al.[7]) are plotted for comparison.

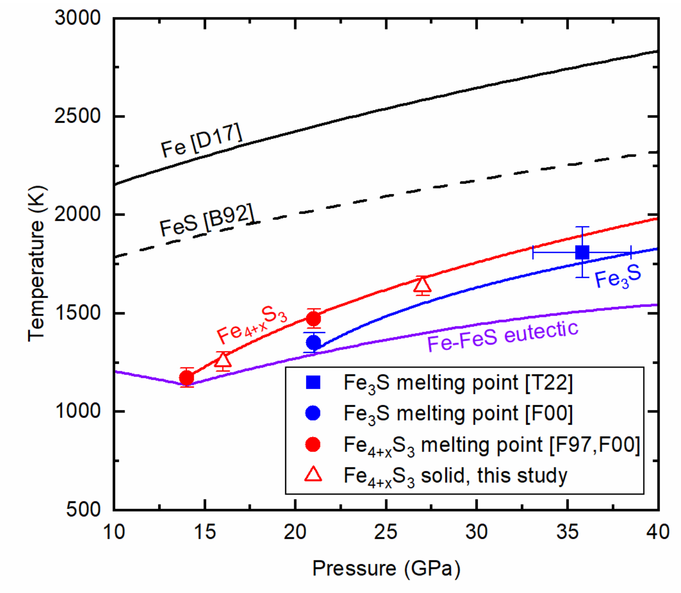

Supplementary Fig. 4 The melting curves of $Fe_{4+x}S_3$ and Fe_3S under Martian core conditions evaluated in this study. The red solid curve indicates the melting temperature of $Fe_{4+x}S_3$, constrained by data from this study (red open triangles) and the literature (red circles[8,9]) where the liquidus phase was originally reported as Fe_3S_2 or $Fe_{3+x}S_2$. The red open triangles indicate the temperature at which $Fe_{4+x}S_3$ coexists with Fe-S liquids, which constrains the lower limit of $Fe_{4+x}S_3$ melting. The blue solid curves indicate the melting curves of Fe_3S used in the model in this study. The blue circle[9] and blue square[10] denote the reported melting temperatures of Fe_3S from the literature. For comparison, the melting curves of Fe[11], FeS[12], and the Fe-FeS eutectic temperatures evaluated in this study are also plotted. The error bars for the red open triangles indicate the uncertainty in temperature measurements estimated in this study, while those for the other symbols are based on the reported uncertainties from the original literature[8-10].

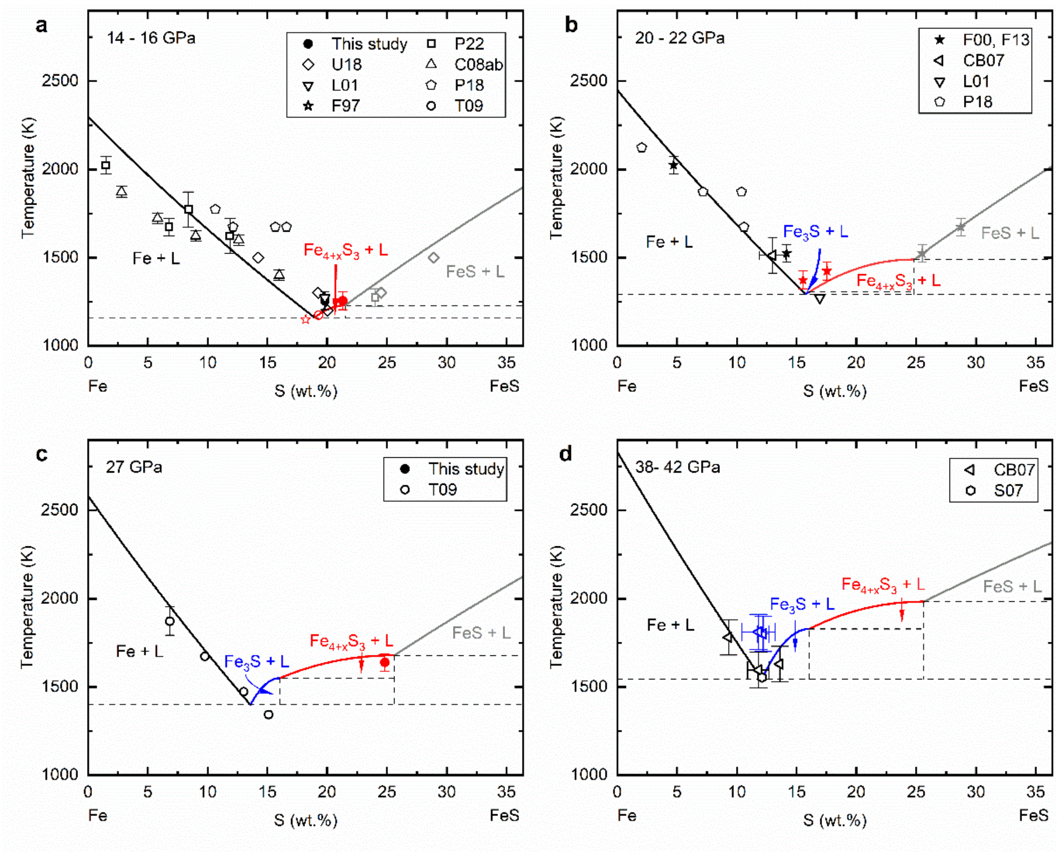

Supplementary Fig. 5 Melting phase diagrams of the Fe-FeS system at 15 GPa (a), 21 GPa (b), 27 GPa (c), and 40 GPa (d). The curves are generated from the model derived in this study, with the colors indicating liquidus curves where particular phases are on the liquidus, black for Fe, blue for Fe_3S, red for $Fe_{4+x}S_3$, and grey for FeS. The liquidus curves where Fe and FeS are liquidus phases are assumed to vary linearly with sulfur concentration, while those with Fe_3S and $Fe_{4+x}S_3$ as the liquidus phases are assumed to follow a parabolic relationship. The solid circles are the experimental data from this study. The symbols P22[13], U18[14], C08ab[15-16], L01[17], P18[18], F97[8], T09[19], F00[9], F13[20], CB07[21], and S07[22] indicate the corresponding literature. The error bars for the data in this study reflect the estimated uncertainties in temperature measurements, while those for the other symbols are based on the reported uncertainties from the original literature.

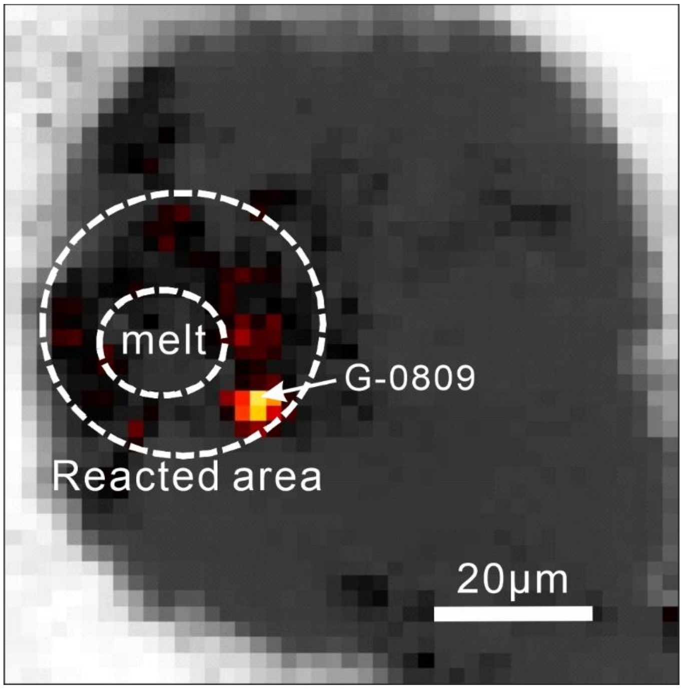

Supplementary Fig. 6 Two-dimensional mapping of the (2 1 3) Peak of $Fe_{4+x}S_3$ in Experiment LJFeS01. This diffraction map was created by systematically moving the sample stage in 2 μm increments. The specific location chosen for the single XRD measurement is highlighted in the image and labeled as G-0809.

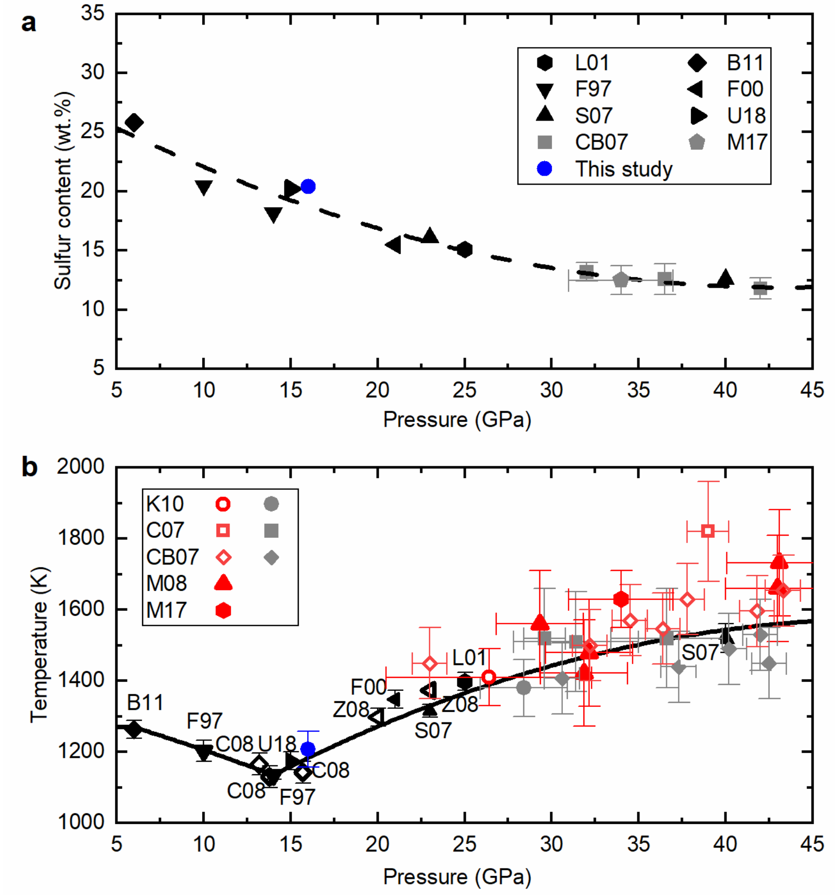

Supplementary Fig. 7 Eutectic compositions (a) and eutectic temperatures (b) in the Fe-FeS system as a function of pressure. The blue circles indicate the data from this study. Data points denoted L01[17], S07[22], F97[8], B11[23], F00[9], CB07[21], K10[24], C07[25], M08[26], and M17[27] are taken from the corresponding literature. In Fig. b, black and blue symbols indicate the eutectic temperatures, while red and grey symbols indicate temperatures where melts and solids are detected, respectively. The error bars for the data in this study reflect the estimated uncertainties in temperature measurements, while those for the other symbols are based on the reported uncertainties from the original literature.

Supplementary Table 1. Experimental Conditions and Identified $Fe_{3+x}S_2$ in LH-DAC Runs.

Run No.	Starting Material	Pressure Medium	P (GPa)*	T (K)†	Unit Cell Volume at 300 K	x‡
LJFeS01	$Fe_{4+x}S_2$ (S7995)	KCl	14.9(1)	1150(200)	324.0(6)	0.11(1)
LD101	$Fe_{4+x}S_2$ (I1570a)	He	21.1(5)	1400(200)	331.2(2)	0.77(1)

*The pressures are measured at room temperature after laser heating. KCl was used as a pressure standard[28] in run LJFeS01. The Raman shift of diamond culets[29] was used to estimate the pressure of LD101 at room temperature.

†In run LJFeS01, the temperatures were not directly measured but estimated based on the phase relations and compositions of the run products. For run LD101, the temperature was determined by fitting the collected thermal radiation spectrum with the Planck radiation function, following the gray-body approximation.

‡The x values in $Fe_{4+x}S_3$ were estimated by single crystal XRD refinements.

Supplementary Table 2. Crystallographic data and structure refinement details for $Fe_{4+x}S_3$

	**$Fe_{4.11}S_3$**	**$Fe_{4.77}S_3$**
**Formula weight**	325.72	362.58
**Pressure**	14.9(1) GPa	21.5(5) GPa
**Temperature**	298(2) K	298(2) K
**Wavelength**	0.410 Å	0.410 Å
**Crystal system**	Orthorhombic	Orthorhombic
**Space group**	Pnma	Pnma
**Unit cell dimensions**	*a* = 10.897(5) Å α = 90°<br>*b* = 3.1252(6) Å β = 90°<br>*c* = 9.515(18) Å γ = 90°	*a* = 11.073(3) Å α = 90°<br>*b* = 3.1820(6) Å β = 90°<br>*c* = 9.435(4) Å γ = 90°
**Volume**	324.0(6) Å^3	332.43(17) Å^3
**Z**	4	4
**Density (calculated)**	6.671 g/cm^3	7.245 g/cm^3
**Absorption coefficient**	4.18 mm^{-1}	4.67 mm^{-1}
**F(000)**	619	688
**Crystal size**	0.015 x 0.013 x 0.013 mm^3	0.003 x 0.002 x 0.002 mm^3
**θ range for data collection**	3.96 to 21.00°	1.64 to 15.40°
**Index ranges**	-14<=*h*<=14, -4<=*k*<=4, -6<=*l*<=7	-13<=*h*<=11, -4<=*k*<=3, -9<=*l*<=8
**Reflections collected**	259	252
**Independent reflections**	204 [R(int) = 0.020]	178 [R(int) = 0.042]
**Coverage of independent reflec-tions**	39.4%	58.1%
**Refinement method**	Full-matrix least-squares on F^2	Full-matrix least-squares on F^2
**Data / restraints / parameters**	204 / 6 / 47	178 / 0 / 46
**Goodness-of-fit**	1.157	1.017
**Final R indices [>2σ(I)]**	R_{obs} = 0.058, wR_{obs} = 0.157	Robs = 0.055, wR_{obs} = 0.138
**R indices [all data]**	R_{all} = 0.073, wR_{all} = 0.192	Rall = 0.075, wR_{all} = 0.152
**Largest diff. peak and hole**	0.86 and -1.02 e·Å^{-3}	1.87 and -1.25 e·Å^{-3}

Supplementary Table 3. Unit Cell Parameters of $Fe_{4+x}S_3$ at High Pressures and 300 K measured in DAC.

Pressure (GPa)*	*a* (Å)	*b* (Å)	*c* (Å)	*V* (Å^3)
**LJFeS01, x = 0.11**				
1.6(1)	11.466(2)	3.245(4)	9.737(1)	362.3(6)
14.8(6)	10.929(1)	3.165(1)	9.420(7)	325.9(3)
14.9(1)	10.897(5)	3.125(1)	9.515(2)	324.2(3)
15.4(4)	10.991(7)	3.160(3)	9.319(1)	323.6(6)
16.1(6)	10.944(8)	3.159(3)	9.279(1)	320.7(4)
17.1(9)	10.953(8)	3.133(3)	9.316(1)	319.6(6)
19.4(7)	10.812(1)	3.138(1)	9.304(8)	316.2(3)
21.3(10)	10.787(3)	3.129(3)	9.261(2)	312.6(6)
22.5(11)	10.766(2)	3.120(1)	9.284(8)	311.8(3)
**LD101, x = 0.77**				
20.1(5)	11.054(3)	3.178(7)	9.428(5)	331.2(2)

*The pressures in LJFeS01 were determined based on the KCl pressure scale[28] and the pressure in LD101 was estimated by the Raman shift of diamond anvils[29].

Supplementary Table 4. Unit Cell Parameters of $Fe_{4+x}S_3$ from Synchrotron Multi-anvil Experiments.

Run No./ File No.	S. M. (wt.%)	P (GPa)	T† (K)	Unit Cell Parameters of $Fe_{4+x}S_3$			
				a (Å)	*b* (Å)	*c* (Å)	*V* (Å^3)
**PEISCHE, SOLEIL**							
**MA231**/017	$Fe_{85}S_{15}$	13.4(5)	1100(50)	11.278(3)	3.217(1)	9.597(3)	348.2(1)
**MA233**/016	$Fe_{85}S_{15}$	13.9(5)	800(50)	11.072(2)	3.152(1)	9.494(2)	331.3(1)
**MA233**/017	$Fe_{85}S_{15}$	14.9(5)	1100(50)	11.277(1)	3.217(1)	9.656(1)	350.2(1)

*The pressures are determined based on the Al_2O_3 pressure scale[30].
†Temperatures were measured using a type-D thermocouple, with the pressure effects on thermocouple EMF corrected[31].
S.M. is starting material

Supplementary Table 5. Chemical Compositions of Run Products in Multi-anvil Experiments.

Run No.	P* (GPa)	T* (K)	Starting Material (wt.%)	Duration (min)	Phase	Compositions determined by EPMA†				
						Fe (wt.%)	S (wt.%)	O (wt.%)	Total (wt.%)	N
S7995	14	918	$Fe_{72.3}S_{27.7}$	210	$Fe_{4+x}S_3$	70.0(2)	29.5(2)	0.3(1)	99.8(3)	17
					FeS	62.9(1)	36.3(1)	0.2(0)	99.3(2)	6
H5840a	16	995	$Fe_{87.5}S_{12.5}$	600	$Fe_{4+x}S_3$	70.7(3)	28.6(2)	0.6(1)	100.0(4)	12
					Fe	99.3(3)	0.1(0)	0.4(1)	99.8(3)	8
H5840b	16	995	$Fe_{80}S_{20}$	600	$Fe_{4+x}S_3$	70.7(3)	28.7(2)	0.6(0)	100.0(3)	6
					Fe	99.2(2)	0.1(0)	0.3(1)	99.5(2)	7
H5840c	16	995	$Fe_{75}S_{25}$	600	$Fe_{4+x}S_3$	70.6(3)	28.5(3)	0.6(0)	99.7(2)	9
					Fe	99.4(2)	0.1(1)	0.3(0)	99.7(2)	5
H5840d	16	995	$Fe_{70}S_{30}$	600	$Fe_{4+x}S_3$	69.9(2)	29.4(1)	0.7(1)	99.9(2)	18
					FeS	62.8(1)	35.9(2)	0.2(1)	98.9(2)	9
S8013a	16	1161	$Fe_{87.5}S_{12.5}$	210	$Fe_{4+x}S_3$	71.4(2)	28.0(2)	0.6(1)	99.9(2)	10
					Fe	98.7(3)	0.6(2)	0.4(1)	99.7(2)	15
S8013b	16	1161	$Fe_{80}S_{20}$	210	$Fe_{4+x}S_3$	71.3(2)	27.9(2)	0.4(0)	99.6(2)	11
					Fe	98.7(3)	0.3(2)	0.2(0)	99.3(2)	9
S8013c	16	1161	$Fe_{75}S_{25}$	210	$Fe_{4+x}S_3$	71.2(2)	27.8(2)	0.4(0)	99.5(2)	9
					Fe	98.2(5)	0.6(2)	0.3(1)	99.1(3)	8
S8013d	16	1161	$Fe_{70}S_{30}$	210	$Fe_{4+x}S_3$	70.8(3)	28.2(1)	0.6(1)	99.6(4)	18
					FeS	62.9(2)	35.9(1)	0.3(0)	99.1(3)	9
H5837a	16	1266	$Fe_{87.5}S_{12.5}$	180	$Fe_{4+x}S_3$	71.5(3)	27.6(2)	0.5(1)	99.5(3)	12
					Fe	97.6(2)	2.0(0)	0.2(0)	99.8(2)	9
					melt	79.4(2)	19.6(1)	0.3(1)	99.3(2)	32
H5837b	16	1266	$Fe_{80}S_{20}$	180	$Fe_{4+x}S_3$	71.6(3)	27.8(2)	0.4(1)	99.7(3)	38
					Fe	97.3(3)	1.7(1)	0.2(0)	99.2(2)	7
					melt	79.1(2)	19.6(1)	0.4(1)	99.1(2)	15
H5837c	16	1266	$Fe_{75}S_{25}$	180	$Fe_{4+x}S_3$	71.4(2)	27.8(2)	0.4(1)	99.6(3)	26
					melt	77.9(2)	21.2(2)	0.4(0)	99.4(1)	24
H5837d	16	1266	$Fe_{70}S_{30}$	180	$Fe_{4+x}S_3$	71.0(2)	28.3(2)	0.4(1)	99.8(2)	12
					FeS	62.6(2)	36.2(2)	0.2(1)	99.1(3)	15
					melt	77.5(3)	21.4(3)	0.5(1)	99.4(1)	5
I1691a	27	1640	$Fe_{75}S_{25}$	30	$Fe_{4+x}S_3$	73.2(2)	26.2(1)	0.6(1)	100.0(2)	13
					melt	74.2(2)	24.5(3)	0.8(1)	99.4(1)	16

*The uncertainties of pressures and temperatures are estimated to be 2 GPa and 50 K, respectively. Temperatures were measured using a type-D thermocouple, with the pressure effects on thermocouple EMF corrected[31].
†The figures within the brackets represent the standard deviations, expressed in the smallest cited units, and 'N' denotes the number of analyses.

Supplementary Table 6. Parameters describing the melting phase diagram of the Fe-FeS system

Parameter	Expression/ Reference
Melting temperature of Fe	Dorogokupets et al.[11]
Melting temperature of FeS	Boehler[12]
Melting temperature of Fe_3S (K)*	T_0 = 1305, P_0 = 21, a = 4.6(5), c = 4.8(5)
Melting temperature of $Fe_{4+x}S_3$ (K)*	T_0 = 1173, P_0 = 14, a = 6.5(5), c = 3.1(3)
Eutectic temperature (K)	$T_{eu} = 1135 + 26.6 \times (P - 14) - 0.44 \times (P - 14)^2$, $\boldsymbol{P \geq 14}$ **GPa**
Eutectic composition (wt.% S)	$X_{eu} = 31.1 - 1.03 \times P + 0.014 \times P^2$
Peritectic composition (wt.% S) (Fe_3S to $Fe_{4+x}S_3$)	$X_{per1} = 16.1$
Peritectic composition (wt.% S) ($Fe_{4+x}S_3$ to FeS)	$X_{per2} = \begin{cases} 0.55 \times (P - 14) + 21.0, \mathbf{14} \leq \boldsymbol{P} \leq \mathbf{22.4\ GPa} \\ 25.6, \boldsymbol{P} > \mathbf{22.4\ GPa} \end{cases}$

*The melting temperature of Fe_3S and $Fe_{4+x}S_3$ are expressed using the Simon-Glatzel equation[32]: $T_{met} = T_0 \left(\frac{P - P_0}{a}\right)^{1/c}$, where P and P_0 are in GPa and T_0 in K.

Supplementary References

1. Dolomanov, O. V., Bourhis, L. J., Gildea, R. J., Howard, J. A., & Puschmann, H. OLEX2: a complete structure solution, refinement and analysis program. J. Appl. Crystallogr. **42**, 339-341 (2009).

2. Tsujino, N., Nishihara, Y., Nakajima, Y., Takahashi, E., Funakoshi, K.I. & Higo, Y. Equation of state of γ-Fe: Reference density for planetary cores. Earth Planet. Sci. Lett. **375**, 244-253 (2013).

3. Thompson, S., Komabayashi, T., Breton, H., Suehiro, S., Glazyrin, K., Pakhomova, A., & Ohishi, Y. Compression experiments to 126 GPa and 2500 K and thermal equation of state of Fe_3S: Implications for sulphur in the Earth's core. Earth Planet. Sci. Lett. **534**, 116080 (2020).

4. Urakawa, S., Someya, K., Terasaki, H., Katsura, T., Yokoshi, S., Funakoshi, K. I., Utsumi, W., Katayama, Y., Sueda, Y.I. & Irifune, T. Phase relationships and equations of state for FeS at high pressures and temperatures and implications for the internal structure of Mars. Phys. Earth Planet. Inter. **143**, 469-479 (2004).

5. Samuel, H., Drilleau, M., Rivoldini, A., Xu, Z., Huang, Q., Garcia, R. F., Lekić, V., Irving, J. C., Badro, J., Lognonné, P. H. & Connolly, J. A. Geophysical evidence for an enriched molten silicate layer above Mars's core. Nature **622**, 712-717 (2023).

6. Irving, J.C., Lekić, V., Durán, C., Drilleau, M., Kim, D., Rivoldini, A., Khan, A., Samuel, H., Antonangeli, D., Banerdt, W. B. & Beghein, C. First observations of core-transiting seismic phases on Mars. Proc. Natl. Acad. Sci. U.S.A. **120**, e2217090120 (2023).

7. Khan, A., Huang, D., Durán, C., Sossi, P. A., Giardini, D. & Murakami, M. Evidence for a liquid silicate layer atop the Martian core. Nature, **622**, 718-723 (2023).

8. Fei, Y., Bertka, C. M. & Finger, L. W. High-pressure iron-sulfur compound, Fe_3S_2, and melting relations in the Fe-FeS system. Science, **275**, 1621-1623 (1997).

9. Fei, Y., Li, J., Bertka, C. M. & Prewitt, C. T. Structure type and bulk modulus of Fe_3S, a new iron-sulfur compound. Am. Mineral. **85**, 1830-1833 (2000).

10. Thompson, S., Sugimura-Komabayashi, E., Komabayashi, T., McGuire, C., Breton, H., Suehiro, S. & Ohishi, Y. High-pressure melting experiments of Fe_3S and a thermodynamic model of the Fe–S liquids for the Earth's core. J. Phys. Condens. Matter. **34**, 394003 (2022).

11. Dorogokupets, P. I., Dymshits, A. M., Litasov, K. D. & Sokolova, T. S. Thermodynamics and equations of state of iron to 350 GPa and 6000 K. Sci. Rep. **7**, 41863 (2017).

12. Boehler, R. Melting of the Fe-FeO and the Fe-FeS systems at high pressure: Constraints on core temperatures. Earth Planet. Sci. Lett. **111**, 217-227 (1992).

13. Pease, A. & Li, J. Liquidus determination of the Fe-S and (Fe, Ni)-S systems at 14 and 24 GPa: Implications for the Mercurian core. Earth Planet. Sci. Lett. **599**, 117865 (2022).

14. Urakawa, S., Kamuro, R., Suzuki, A. & Kikegawa, T. Phase relationships of the system Fe-Ni-S and structure of the high-pressure phase of $(Fe_{1-x}Ni_x)_3S_2$. Phys. Earth Planet. Inter. **277**, 30-37 (2018).

15. Chen, B., Gao, L., Leinenweber, K., Wang, Y., Sanehira, T. & Li, J. In situ investigation of high-pressure melting behavior in the Fe-S system using synchrotron X-ray radiography. High Pressure Research **28**, 315-326 (2008).

16. Chen, B., Li, J. & Hauck, S. A. Non-ideal liquidus curve in the Fe-S system and Mercury's snowing core. Geophys. Res. Lett. **35**, L07201 (2008).

17. Li, J., Fei, Y., Mao, H.K., Hirose, K. & Shieh, S. R. Sulfur in the Earth's inner core. Earth Planet. Sci. Lett. **193**, 509-514 (2001).

18. Pommier, A., Laurenz, V., Davies, C. J. & Frost, D. J. Melting phase relations in the Fe-S and Fe-S-O systems at core conditions in small terrestrial bodies. Icarus **306**, 150-162 (2018).

19. Tsuno, K. & Ohtani, E. Eutectic temperatures and melting relations in the Fe–O–S system at high pressures and temperatures. Phys. Chem. Miner. **36**, 9-17 (2009).

20. Fei, Y. Simulation of the planetary interior differentiation processes in the laboratory. J. Vis. Exp. **81**, e50778 (2013).

21. Chudinovskikh, L. & Boehler, R. Eutectic melting in the system Fe–S to 44 GPa. Earth Planet. Sci. Lett. **257**, 97-103 (2007).

22. Stewart, A. J., Schmidt, M. W., Van Westrenen, W. & Liebske, C. Mars: A new core-crystallization regime. Science **316**, 1323-1325 (2007).

23. Buono, A. S. & Walker, D. The Fe-rich liquidus in the Fe–FeS system from 1 bar to 10 GPa. Geochim. Cosmochim. Acta **75**, 2072-2087 (2011).

24. Kamada, S., Terasaki, H., Ohtani, E., Sakai, T., Kikegawa, T., Ohishi, Y., Hirao, N., Sata, N. & Kondo, T. Phase relationships of the Fe–FeS system in conditions up to the Earth's outer core. Earth Planet. Sci. Lett. **294**, 94-100 (2010).

25. Campbell, A. J., Seagle, C. T., Heinz, D. L., Shen, G. & Prakapenka, V. B. Partial melting in the iron–sulfur system at high pressure: A synchrotron X-ray diffraction study. Phys. Earth Planet. Inter. **162**, 119-128 (2007).

26. Morard, G., Andrault, D., Guignot, N., Sanloup, C., Mezouar, M., Petitgirard, S. & Fiquet, G. In situ determination of Fe–Fe_3S phase diagram and liquid structural properties up to 65 GPa. Earth Planet. Sci. Lett. **272**, 620-626 (2008).

27. Mori, Y., Ozawa, H., Hirose, K., Sinmyo, R., Tateno, S., Morard, G. & Ohishi, Y. Melting experiments on Fe–Fe_3S system to 254 GPa. Earth Planet. Sci. Lett. **464** 135-141 (2017).

28. Dewaele, A., Belonoshko, A. B., Garbarino, G., Occelli, F., Bouvier, P., Hanfland, M. & Mezouar, M. High-pressure–high-temperature equation of state of KCl and KBr. Phys. Rev. B **8**, 214105 (2012).

29. Akahama, Y. & Kawamura, H. Pressure calibration of diamond anvil Raman gauge to 310 GPa. J. Appl. Phys. **100**, 043516 (2006).

30. Shi, W., Wei, W., Sun, N., Mao, Z. & Prakapenka, V. B. Thermal Equations of State of Corundum and Rh_2O_3 (II) ‐ Type Al_2O_3 up to 153 GPa and 3400 K. J. Geophys. Res.: Solid Earth **127**, e2021JB023805 (2022).

31. Nishihara, Y., Doi, S., Kakizawa, S., Higo, Y. & Tange, Y. Effect of pressure on temperature measurements using WRe thermocouple and its geophysical impact. Phys. Earth Planet. Inter. **298**, 106348 (2020).

32. Simon, F. & Glatzel, G. Bemerkungen zur schmelzdruckkurve. Z. Anorg. Allg. Chem. **178**, 309-316 (1929).
